# Circulating Oxidized mtDNA is Associated Broadly with Cardiovascular Disease in a Longitudinal Cohort Study of Psoriasis

**DOI:** 10.1016/j.xjidi.2023.100243

**Published:** 2023-11-03

**Authors:** Sundus S. Lateef, Grace A. Ward, Haiou Li, Carla Pantoja, Elizabeth Florida, Christin G. Hong, Justin Rodante, Andrew Keel, Marcus Y. Chen, Alexander V. Sorokin, Martin P. Playford, Nehal N. Mehta

**Affiliations:** 1 Section of Inflammation and Cardiometabolic Diseases, National Heart, Lung, and Blood Institute, National Institutes of Health, Bethesda, Maryland, USA; 2 Department of Immunology, St. Jude Children's Research Hospital, Memphis, Tennessee, USA

**Keywords:** Cardiovascular disease, IL-17 signaling in psoriasis, Oxidized mtDNA

## Abstract

Psoriasis (PSO) is a chronic and systemic inflammatory autoimmune disease associated with atherosclerosis and myocardial infarction. Given that atherosclerosis is both inflammation and immune driven, we sought to expand on known immune and inflammatory biomarkers in a PSO cohort. In this study, we focus on oxidized mtDNA (ox-mtDNA), a product of cells undergoing pyroptosis, including keratinocytes, which was quantified in patients with PSO and individuals without PSO by ELISA. Patients with PSO had significantly higher ox-mtDNA levels than healthy subjects (mean ± SD = 9246 ± 2518 pg/ml for patients with PSO vs 7382 ± 2506 pg/ml for those without; *P* = .006). Importantly, ox-mtDNA was positively associated with IL-17a (β = 0.25; *P* = .03) and low-density granulocytes (β = 0.37; *P* = .005) but negatively associated with high-density lipoprotein-cholesterol (β = −0.29; *P* = .006). After adjusting for traditional cardiovascular risk factors, we found that ox-mtDNA was associated with noncalcified coronary burden, which was measured by coronary computed tomography angiography (β = 0.19; *P* = .003). Biologic-naïve patients with PSO receiving anti–IL-17a therapy had a 14% decrease in ox-mtDNA (mean ± SD: 10540 ± 614 pg/ml at baseline to 9016 ± 477 pg/ml at 1 year; *P* = .016) and a 10% reduction in noncalcified coronary artery burden (mean ± SD: 1.06 ± 0.45 at baseline, reducing to 0.95 ± 0.35 at 1 year; *P* = .0037). In summary, levels of ox-mtDNA in PSO are associated with measures of coronary plaque formation, indicating that this biomarker may be an autoimmune-driven early atherosclerotic feature.

## Introduction

Myocardial infarction is the leading cause of death worldwide (Global Burden of Metabolic Risk Factors for Chronic Diseases, 2014). Atherosclerosis, the primary cause of myocardial infarction, is a disease driven by lipid infiltration, inflammatory cell activation, and metabolic derangement (Libby et al, 2009). To better understand the link between chronic inflammatory states and atherosclerosis, we have utilized psoriasis (PSO) as a human model for the study of coronary artery disease (CAD). PSO is a chronic inflammatory disease with increased cardiovascular risk, including subclinical atherosclerosis (Elnabawi et al, 2019b; Mehta et al, 2010). Despite being younger than patients with traditional cardiovascular risk factors, patients with PSO have a higher prevalence of atherosclerotic plaque (Lerman et al, 2017). Furthermore, atherosclerosis, particularly the development of CAD, is associated with PSO severity (Baumer et al, 2018; Elnabawi et al, 2019a; Lerman et al, 2017); yet, the underlying mechanisms are not completely understood.

We previously reported that in PSO, a unique subpopulation of neutrophils (low-density granulocytes [LDGs]) is upregulated in circulation compared with that in controls, and the frequency of LDGs correlated with CAD as assessed by coronary computed tomography angiography (CCTA) (Teague et al, 2019). LDGs have elevated mitochondrial superoxide production, and the DNA released by neutrophil extracellular traps (NETs) is highly enriched in oxidized mtDNA (ox-mtDNA), which enhances the formation of NETs and triggers the inflammasome, a macromolecular complex that directs innate immune activation, within neutrophils (Lood et al, 2016). Notably, ox-mtDNA is implicated in the pathogenesis of atherosclerosis (Nakayama and Otsu, 2018). In atherogenic mouse models, inflammasome activation is associated with NET formation within nascent atherosclerotic plaques (Westerterp et al, 2018).

Taken together, we hypothesized that elevated circulating ox-mtDNA would be (i) observed in patients with PSO, (ii) associated with measures of immune dysfunction (as assessed by LDGs), and (iii) associated with noncalcified coronary plaque burden (a CCTA marker of subclinical atherosclerosis).

## Results

### Study population

Patients with PSO and control subjects were young (Table 1) (mean ± SD = 49.9 ± 12.5 years for patients with PSO vs 45.1 ± 13.5 years for control subjects; *P* = .19) and were mostly male (Table 1) (n = 47 [64%] for patients with PSO vs 11 [79%] for control subjects; *P* = .29), with overweight body mass index (Table 1) (median = 27.8 and interquartile range [IQR] = 24.0–31.6) noted only in patients with PSO. Both patients with PSO (mean PASI score = 7.8 ± 5.7) and control subjects had a similar low cardiovascular risk by Framingham risk score (Table 1) (median [IQR] = 1.81 [0.59–4.08] in patients with PSO vs 0.70 [0.10–2.10] in control subjects; *P* = .09), likely because of the young age of both study populations.

**Table 1:** Baseline Characteristics of Patients with Psoriasis and Controls

Variable	Psoriasis (n = 89)	Controls (n = 14)	*P*-Value
Demographic and clinical characteristics			
Age, y	49.9 ± 12.5	45.1 ± 13.5	.190
Males, n^1^	47 (64)	11 (79)	.290
Hypertension, n (%)	25 (28)	2 (14)	.280
Hyperlipidemia	36 (40)	3 (21)	.170
Type-2 diabetes	9 (10)	0 (0)	.210
Body mass index	27.8 (24.0–31.6)	25.1 (23.2–27.0)	.060
Current smoker	7 (8)	2 (14)	.430
Statin use	22 (25)	1 (7)	.140
Clinical and laboratory values			
Total cholesterol, mg/dl	174 (153–202)	168 (152–204)	.900
HDL cholesterol, mg/dl	52 (46–64)	60 (49–78)	.120
LDL cholesterol, mg/dl	98 (83–118)	83 (70–120)	.370
Apolipoprotein A1	147 (136–164)	147 (142–187)	.270
Apolipoprotein B	88 (75–102)	77 (60–110)	.270
Triglycerides, mg/dl	93 (68–121)	91 (79–110)	.880
Cholesterol efflux capacity	0.926 (0.837–1.062)	1.047 (0.887–1.285)	.060
Framingham risk score	1.81 (0.59–4.08)	0.70 (0.10–2.10)	.090
Inflammatory markers			
GlycA, μmol/l	392 (359–451)	341 (332–375)	.003
IL-1β, pg/ml	0.13 (0.04–0.17)	0.04 (0.02–0.07)	.060
IL-6, pg/ml	1.39 (0.80–2.33)	0.70 (0.44–0.92)	.002
IL-17a, pg/ml	1.51 (0.82–3.08)	0.63 (0.20–0.92)	.001
TNF-α, pg/ml	1.34 (0.8–2.12)	1.27 (1.19–1.44)	.78
Low-density granulocytes	51.4 (23.0–99.7)	35.3 (21.6–228.5)	.91
Normal-density granulocytes	3617 (3030–4312)	3212 (2258–3639)	.16
ox-mtDNA, pg/ml	9246 ± 2518	7382 ± 2506	.006
ox-mtDNA/glucose, pg/ml	142 ± 44	103 ± 46	.003

Abbreviations: HDL, high-density lipoprotein; IQR, interquartile range; LDL, low-density lipoprotein; ox-mtDNA, oxidized mtDNA.

Values are reported as mean ± SD or median (IQR) for continuous data and n (%) for categorical data. Continuous data were compared using *t*-test for parametric and Wilcoxon rank-sum for nonparametric observations. Groups containing categorical data were compared using Pearson’s chi-square test.

1 Patients self reported.

### ox-mtDNA levels in PSO versus in controls

Circulating ox-mtDNA was significantly higher in the PSO subpopulation than in the control subpopulation (Table 1) (mean ± SD = 9246 ± 2518 pg/ml in PSO vs 7382 ± 2506 pg/ml in controls; *P* = .006). As expected, there were also increased inflammatory markers in PSO, including GlycA (Table 1) (median [IQR] = 392 [359–451] in patients with PSO vs 341 [332–375] for the controls; *P* = .003), IL-6 (Table 1) (median [IQR] = 1.39 [0.80–2.33] for patients with PSO vs 0.70 [0.44–0.92] for controls; *P* = .002), and IL-17a (Table 1) (median [IQR] = 1.51 [0.82–3.08] for patients with PSO vs 0.63 [0.20–0.92] for controls; *P* = .001). Thus, as expected, ox-mtDNA and other markers of inflammation are significantly increased in our PSO cohort.

### ox-mtDNA was associated with measures of lipid and metabolic dysfunction

ox-mtDNA did not associate with age (Table 2) (β = 0.02; *P* = .87) but did associate with male gender (Table 2) (β = 0.32; *P* = .002), hypertension (Table 2) (β = 0.25; *P* = .02), body mass index (Table 2) (β = 0.27; *P* = .01), and Framingham risk score (Table 2) (β = 0.24; *P* = .03). There was an association between ox-mtDNA and measures of lipid dysfunction, including negative associations between ox-mtDNA and high-density lipoprotein cholesterol (Table 2) (β = −0.29; *P* = .006) and apolipoprotein A1 (Table 2) (β = −0.25; *P* = .02). There was a positive association between ox-mtDNA and triglycerides (Table 2) (β = 0.29; *P* = .005). Ox-mtDNA was associated with measures of metabolic dysfunction, including a positive association between ox-mtDNA and apolipoprotein B (Table 2) (β = 0.30; *P* = .004), an atherogenic lipid species. Taken together, these data demonstrate that ox-mtDNA tends to be positively correlated with increased early predictors of high cardiovascular risk.

**Table 2:** Association of Oxidized mtDNA with Other Covariates in Psoriasis (n = 89)

Variable	β	*P*-Value
Demographic and clinical characteristics		
Age	0.02	.87
Males^1^	0.32	.002
Hypertension	0.25	.02
Hyperlipidemia	0.11	.31
Type-2 diabetes	−0.09	.38
Body mass index	0.27	.01
Current smoker	0.02	.83
Statin use	0.08	.47
Framingham risk score	0.24	.03
Clinical and laboratory values		
Total cholesterol	0.11	.32
HDL cholesterol	−0.29	.006
LDL cholesterol	0.19	.07
Apolipoprotein A1	−0.25	.02
Apolipoprotein B	0.30	.004
Triglycerides	0.29	.005
Cholesterol efflux capacity	−0.18	.1
Psoriasis severity		
PASI score	0.06	.58
Inflammatory markers		
GlycA	0.10	.38
IL-1β	−0.03	.73
IL-6	0.01	.96
IL-17a	0.25	.03
TNF-α	0.003	.98
Low-density granulocytes	0.37	.005
Normal-density granulocytes	0.04	.78
Coronary artery characterization		
Total burden	0.26	< .001
Noncalcified coronary artery burden	0.27	< .001

Abbreviations: HDL, high-density lipoprotein; LDL, low-density lipoprotein.

Data are represented as standardized β-coefficient and *P*-value.

1 Patients self reported.

### ox-mtDNA was associated with measures of inflammatory and immune dysfunction

In addition to lipid dysfunction, markers of immune dysfunction were also associated with ox-mtDNA (Table 2). We noted positive associations between ox-mtDNA and IL-17a (β = 0.25; *P* = .03) as well as with LDGs, a proinflammatory subpopulation of neutrophils (β = 0.37; *P* = .005). In contrast, an association between ox-mtDNA and normal-density granulocytes was not observed (β = 0.04; *P* = .78), suggesting that the increased ox-mtDNA in PSO may be related to the well-established increase in neutrophils, potentially related to NLRP3-mediated NETosis (Shao et al, 2019).

### ox-mtDNA was directly associated with subclinical atherosclerosis

Among patients with PSO, ox-mtDNA was directly associated with CAD as assessed by noncalcified coronary plaque burden (Table 2) (β = 0.27; *P* < .001). This relationship was maintained in models adjusted for traditional cardiovascular risk factors (eg, Framingham risk score, statin therapy, anthropomorphic measure of obesity) and systemic inflammation (eg, PSO severity as assessed by PASI score and high-sensitivity C-reactive protein) (β = 0.19; *P* = .003).

### Longitudinal analysis of ox-mtDNA by biologic treatment type

In our PSO cohort, 13 patients were biologic naïve at baseline but undertook anti–IL-17a therapy (secukinumab or ixekizumab) for 1 year. These patients had a reduction in PSO skin disease severity (median [IQR] PASI = 14.4 [8.6–20.4] at baseline, reducing to 2.4 [0.6–7.9]; *P* < .001) as well as noncalcified coronary plaque burden from baseline to 1 year (mean ± SD = 1.18 ± 0.10 at baseline, reducing to 1.09 ± 0.09 at 1 year; *P* = .0047). There was a significant decrease in PSO skin disease severity (median [IQR] PASI = 7.7 [5.1–13.4] at baseline, reducing to 2.8 [1.8–4.2]; *P* = .003) in patients receiving anti–TNF-α therapy (adalimumab or etanercept) for 1 year and a nonsignificant difference in PSO severity at 1 year in the anti–TNF-α–treated group compared with that in anti–IL-17a–treated group (*P* = .56). However, there was no decrease in noncalcified coronary burden from baseline to 1 year in the patients receiving anti–TNF-α therapy (mean ± SD = 1.28 ± 0.08 at baseline compared with 1.23 ± 0.098 at 1 year; *P* = .19).

**Figure 1:**
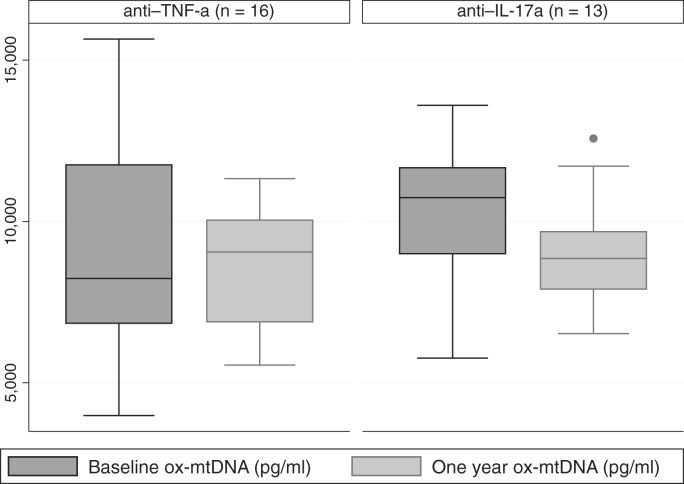
**Change in ox-mtDNA levels over 1 year stratified by biologic treatment type.** Data are presented as median ± interquartile range. Outliers are represented by a single dot (eg, within the 1-year data for the anti–IL-17a group*).* Comparisons were made using paired *t*-test. There was a significant decrease in ox-mtDNA levels over 1 year in the group receiving anti–IL-17a treatment for 1 year (*P* = .016) but not in the group receiving anti–TNF-α treatment for 1 year (*P* = .50). ox-mtDNA, oxidized mtDNA.

We postulated that patients with PSO undergoing anti–IL-17a biologic therapy for 1 year would also exhibit a reduction in circulating ox-mtDNA (Figure 1). Indeed, the patients receiving a year of anti–IL-17a biologic therapy had a 14% decrease in circulating ox-mtDNA (Figure 1) (mean ± SD = 10540 ± 614 pg/ml at baseline to 9016 ± 477 pg/ml at 1 year; *P* = .016). A decrease in circulating ox-mtDNA was also observed in patients undertaking anti–TNF-α therapeutics for 1 year (Figure 1) (mean ± SD = 8554 ± 781 pg/ml at baseline vs 8561 ± 497 pg/ml) at 1-year follow-up, but this decrease was not significant (*P* = .50). In summary, we have discovered that ox-mtDNA is an IL-17a–dependent biomarker in patients with PSO that is associated with CAD.

## Discussion

In this cross-sectional study of PSO, we present 4 key findings: (i) that circulating ox-mtDNA is higher in patients with PSO than in a control subpopulation; (ii) a positive association between ox-mtDNA and both IL-17a and LDG levels; (iii) an inverse association between ox-mtDNA and antiatherogenic markers high-density lipoprotein cholesterol and apolipoprotein A1 but a positive association with markers known to be proatherogenic such as high triglycerides and apolipoprotein B; and (iv) in adjusted models for cardiovascular risk factors and systemic inflammation, ox-mtDNA is strongly associated with noncalcified coronary plaque burden, an imaging marker for CAD.

Although, to our knowledge, previously unreported as a biomarker for PSO, ox-mtDNA has been demonstrated as a biomarker for other inflammatory diseases such as lupus and metabolic syndrome, which have both been recognized as risk factors for cardiovascular diseases (CVDs) (Lood et al, 2016; Ye et al, 2023). Elevated circulating ox-mtDNA has also been seen in malignancies such as myelodysplastic syndrome, which is distinct among other myeloid malignancies for exhibiting elevated inflammation in the bone marrow microenvironment (Ward et al, 2021). This elevated inflammation is driven by NLRP3 inflammasome assembly and pyroptotic cell death (Ward et al, 2021). Furthermore, the authors also reported a positive association between ox-mtDNA and members of the alarmin family of proteins, S100A8 and A9. (Ward et al, 2021). In myelodysplastic syndrome, ox-mtDNA contributes to feed-forward bone marrow failure through toll-like receptor 9, an endosomal DNA-sensing pattern recognition receptor known to prime and activate the inflammasome propagating the type 1 IFN–induced inflammatory response in neighboring healthy hematopoietic stem and progenitor cells (Ward et al, 2023).

Although we have yet to investigate inflammasome assembly and pyroptosis in PSO, enhanced inflammasome activity has been reported by other groups (Garshick et al, 2019; Verma et al, 2021). In addition, dysregulation of both pyroptotic and ferroptotic pathways has been observed in psoriatic patients (Zhang et al, 2022). The inflammasome is activated in PSO as measured through transcriptomic studies of caspase-5 and IL-1β and proteomic studies of IL-6 within vascular endothelial cells (Garshick et al, 2019). We have previously demonstrated that the S100A8/A9 dimer (calprotectin) is increased in the serum of patients with PSO (Naik et al, 2015), and in the same cohort, we have also found elevations of circulating S100A7, A8, and A12 (Playford MP, unpublished data). An association between plasma ox-mtDNA/glucose ratio has also been observed with S100A9, S100A12, and the S100A8/A9 dimer (Florida et al, unpublished data).

LDGs are a subpopulation of neutrophils that increase NET release (NETosis) and subclinical atherosclerosis as measured by noncalcified coronary plaque burden in inflammatory diseases (Lood et al, 2016; Teague et al, 2019). In this study, we identified an association between ox-mtDNA, IL-17a, and LDGs, potentially linking IL-17a signaling with the release of oxidized nucleic acid species into NETs produced by LDGs. IL-17a also promotes expression of S100A8 and S100A9 during the inflammatory response of keratinocytes (Christmann et al, 2021). We also show that anti–IL-17a intervention significantly reduces circulating ox-mtDNA (Figure 1); further preliminary data demonstrate that similar therapy also reduces LDGs (Teague et al, unpublished data).

The mechanism by which IL-17a increases oxidation on mtDNA is not known. Although not a cell of myeloid origin, we propose that the HaCaT keratinocyte cell line may be used to assess such a mechanism. To this end, we have pilot studies that show that a modest increase in mitochondrial-oxidized nucleoids was observed with IL-17a but not with TNF-α treatment (Pantoja, unpublished data).

Recent data have proposed an IL-17a–extracellular signal–regulated kinase/NLRP3/caspase-1 signaling pathway leading to pyroptosis in nasal epithelial cells (Li et al, 2022). Pyroptosis is a putative component of the inflammatory response leading to cardiovascular injury, including coronary artery pathology. In previous studies, we have associated CCTA markers of subclinical atherosclerosis, such as rupture-prone noncalcified coronary artery burden and high-risk plaque features, with PSO severity (Lerman et al, 2017). In this study, we noted an association between ox-mtDNA and noncalcified coronary artery burden. Our findings are consistent with those of previous studies of the chronic inflammatory disease PSO and show that ox-mtDNA may be an informative marker to identify patients with PSO who will develop immune, lipid, and metabolic dysfunction as well as early atherosclerosis in PSO.

Our study is limited because it is cross-sectional and does not track serial measurements of the blood marker ox-mtDNA. Our assessment of cardiovascular outcomes would be enhanced by the addition of hard cardiovascular event data. Tracking the incidence of major cardiovascular events such as revascularization, transient ischemic attack, or myocardial infarction would lend to longitudinal analysis but is difficult in our cohort owing to the relatively short duration of the study and the relatively young age of our study population. To further characterize this chronic and systemic inflammatory disease, detailed studies of immune cell populations, including LDGs, and inflammatory processes, particularly the release of NETs and ox-mtDNA, in PSO are needed.

In summary, we have characterized the systemic release of ox-mtDNA and subsequent subclinical atherosclerosis in a large cohort of patients with PSO. ox-mtDNA relates to immune, lipid, and cytokine markers at baseline and modulates with targeted biologic therapy. This work has implications for the inflammatory pathogenesis of autoimmune-driven CVD. Future mechanistic studies must further elucidate these pathways and potential therapeutic inhibitors to target them.

## Materials and Methods

### Demographic and clinical characteristics of PSO and control cohorts

Our study included a cohort of consecutively recruited patients with PSO (n = 89) from January 2013 to November 2019 as part of the Psoriasis, Atherosclerosis, and Cardiometabolic Disease Initiative (NCT01778569) at the National Institutes of Health (NIH) Clinical Center. Participants aged ≥18 years with PSO consisting of typical skin findings and associated joint, nail, and/or hair findings clinically diagnosed by a physician were included in this study. Participants were excluded if they had severe renal excretory dysfunction; estimated glomerular filtration rate <30 ml/min/1.73m2; existing CVD; and any comorbid condition known to be associated with CVD or systemic inflammation, such as uncontrolled hypertension, internal malignancy within 5 years, HIV, active infection within 72 hours of baseline, major surgery within 3 months, and/or pregnancy or lactation. Physician consent was required for donors to enroll in this program, including that they must be healthy per the physician’s standards.

**Figure 2:**
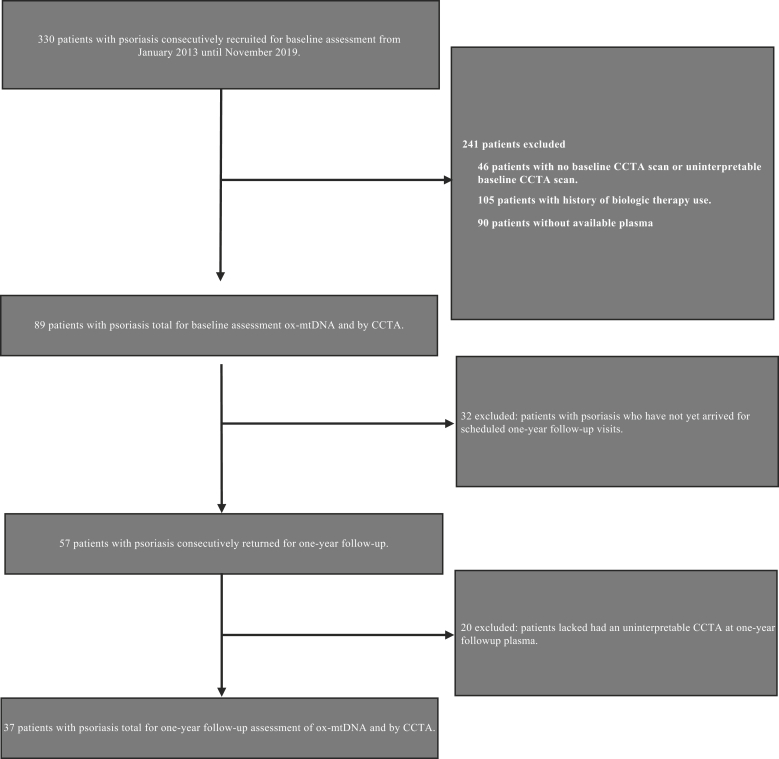
**Recruitment schematic for patients with psoriasis at the National Institutes of Health.** CCTA, coronary computed tomography angiography; ox-mtDNA, oxidized mtDNA.

For comparison, a cohort of healthy volunteers (NCT01934660) (n = 14) was recruited from 2013 to 2019 (Figure 2). Patients were excluded if they had any comorbid condition known to promote CVD or systemic inflammation, such as known CVD, uncontrolled hypertension, internal malignancy within 5 years, HIV, active infection within the past 72 hours of baseline, and major surgery within 3 months. This was a pilot study where consideration of sample size or power was not a priority. To the best of our knowledge, no subjects lacked any of the data points presented. All patients provided written informed consent, and all study protocols were approved by the institutional review board at the NIH. All study protocols are in compliance with the Declaration of Helsinki.

Patients were asked to complete survey-based questionnaires regarding smoking, previous CVD, family history of CVD, and previously established diagnoses of hypertension and diabetes. Patient responses were then confirmed during history and physical examination by the study provider. CVD included acute coronary syndrome comprising both myocardial infarction and unstable angina pectoris; angina pectoris; cerebrovascular event; transient ischemic attack; peripheral vascular disease; and revascularization procedures, including both coronary artery bypass grafting and percutaneous interventional procedures. Diabetes was defined as fasting glucose ≥126 mg/dl, glycated hemoglobin >6.5%, or use of diabetic medication. Hypertension was defined as systolic blood pressure ≥140 mmHg, diastolic blood pressure ≥90 mmHg, or use of antihypertensive medication. Hyperlipidemia was defined as total cholesterol >5.18 mmol/l, low-density lipoprotein cholesterol ≥4.14 mmol/l, or high-density lipoprotein cholesterol ≤1.04 mmol/l. Hypertriglyceridemia was not included in the assessment of hyperlipidemia. A 10-year Framingham risk score was assessed for all patients.

All patients underwent fasting blood draws for the assessment of the lipid panel at baseline, including high- and low-density lipoprotein cholesterol. A dermatologist or physician evaluated all patients with PSO for the assessment of PSO skin disease severity through the PASI score.

### CCTA

All patients underwent blood draw (plasma collection) and CCTA on the same day in the same computed tomography scanner (320-detector row Aquilion ONE ViSION). Guidelines established by the NIH Radiation Exposure Committee were followed. Scans were performed with retrospective gating at 120 kV and tube current of 750–850 mA, with a gantry rotation time ≤420 ms. Image-acquisition characteristics included slice thickness of 0.75 mm and pitch of 0.2–0.4. Coronary artery phenotyping was performed using QAngio CT (Medis Medical Imaging) with high (interclass correlation > 0.95) intraobserver and interobserver agreement and with readers blinded to patient characteristics as previously published (Lerman et al, 2017). Manual adjustment of inner lumen and outer vessel wall delineations was performed if needed. Measurements of noncalcified coronary artery burden (mm^2^) were determined by dividing noncalcified volume (mm^3^) by vessel length (mm) and were attenuated for luminal intensity measures for accuracy. Consecutive patients with PSO were then assessed for change in subclinical atherosclerosis by CCTA at 1-year follow-up.

### Measurements of inflammatory markers

Oxidized DNA was quantified in human plasma using the DNA/RNA oxidative damage (high sensitivity) ELISA kit (Cayman Chemical Company; item number 589320). Plasma glucose concentration, measured by colorimetric assay (Cayman Chemical Company), was performed to control for hyperglycemia-induced inflammasome activation. The indicated cytokines were measured using a custom multiplex assay (Uplex, Meso Scale Diagnostic) following the manufacturer’s instructions.

### Cholesterol efflux capacity

High-density lipoprotein cholesterol efflux capacity assays were with J774 murine macrophage cells. Briefly, 3 × 10^5^ J774 cells/well were seeded in each well of a 24-well plate and radiolabeled with 2 μCi of ^3^H-cholesterol/ml in RPMI-1640 media containing 1% fetal bovine serum for 24 hours. Next, the cells were incubated for 16 hours in RPMI media containing 2% BSA in the presence or absence of 0.3 mmol/l 8-(4-chlorophenylthio)-cAMP to upregulate ABCA1. We added 2.8% apoB-depleted plasma to the efflux medium for 4 hours. To quantify the efflux of radioactive cholesterol from the cells, we used liquid scintillation counting. Efflux was calculated using the following formula: μCi of ^3^H-cholesterol in media containing 2.8% apoB-depleted subject plasma − μCi of ^3^H-cholesterol in plasma-free media/μCi of ^3^H-cholesterol in media containing 2.8% apoB-depleted pooled control plasma − μCi of ^3^H-cholesterol in pooled control plasma-free media. The pooled plasma was obtained from 5 healthy adult volunteers. All assays were performed in duplicate.

### LDGs

Briefly, lysed whole blood cells or ficoll-separated PBMCs were incubated for 30 minutes in a 10-color antibody cocktail and acquired on a BD Biosciences LSRII flow cytometer using DIVA 6.1.2 software (BD Bioscience, San Jose, CA). We determined the frequency of LDGs by quantitating the percentage of CD14^lo^CD15^hi^CD10^hi^ cells in the PBMC fraction by flow cytometry and used the complete blood count to determine the frequency of LDGs per microliter as previously described (Teague et al, 2019).

### Statistical analyses

All data were assessed for normality through skewness and kurtosis. Data were reported as mean with SD for parametric variables, median with IQR for nonparametric variables, and percentages for categorical variables. In baseline analyses, parametric and nonparametric variables were compared between the 2 groups using Student’s *t*-test and Wilcoxon signed-rank test, respectively. In longitudinal analyses, parametric variables were analyzed using paired *t*-test, and nonparametric variables were analyzed using Wilcoxon signed-rank test. Dichotomous variables were analyzed using McNemar’s test for within-group longitudinal analyses. In multivariable linear regression analyses, potential confounding variables were determined by purposeful selection and added to the base model. Standardized beta values from these analyses were reported, which indicate the number of SD changes in the outcome variable per SD change in the predicting variable. *P* < .05 was deemed significant. All statistical analyses were performed using STATA 12 (Stata, College Station, TX) by NIH staff, blinded to clinical demographics and imaging characteristics.

### Ethics statement

Our study included a cohort of consecutively recruited patients with PSO (n = 89) from January 2013 to November 2019 as part of the PSO, Atherosclerosis and Cardiometabolic Initiative (NCT01778569) at the NIH Clinical Center. For comparison, a cohort of healthy volunteers (NCT01934660) (n = 14) was recruited from 2013 to 2019 (Figure 2). All patients provided written informed consent, and all study protocols were approved by the institutional review board at the NIH. All study protocols are in compliance with the Declaration of Helsinki.

### Data availability statement

Data are available upon request. Please contact the corresponding author, MPP, at playfordmp@nhlbi.nih.gov.

## ORCIDs

Haiou Li: http://orcid.org/0000-0002-9057-1346

Elizabeth Florida: http://orcid.org/0000-0001-9128-6160

Carla J. Pantoja: http://orcid.org/0000-0003-3886-3752

Sundus S. Lateef: http://orcid.org/0000-0001-6913-833X

Grace A. Ward: http://orcid.org/0000-0003-4404-7727

Alexander V. Sorokin: http://orcid.org/0000-0002-2291-4888

Marcus Y. Chen: http://orcid.org/0000-0003-0743-9369

Martin P. Playford: http://orcid.org/0000-0002-5571-7266

Nehal N. Mehta: http://orcid.org/0000-0003-4939-5130

Christin G. Hong: http://orcid.org/0000-0002-9705-0746

Justin Rodante: http://orcid.org/0000-0002-2435-7747

Andrew Keel: https://orcid.org/0000-0002-8237-0480

## Conflict of interest

NNM is a full-time United States government employee and has served as a consultant for Amgen, Eli Lilly, and Leo Pharma, receiving grants/other payments; as a principal investigator and/or investigator for AbbVie, Celgene, Janssen Pharmaceuticals, and Novartis, receiving grants and/or research funding; and as a principal investigator for the National Institute of Health, receiving grants and/or research funding. The remaining authors state no conflict of interest.
